# Magnetic resonance imaging–guided conventional catheter ablation of isthmus-dependent atrial flutter using active catheter imaging

**DOI:** 10.1016/j.hroo.2022.06.011

**Published:** 2022-06-30

**Authors:** Stefan Ulbrich, Yan Huo, Jakub Tomala, Michael Wagner, Utz Richter, Liying Pu, Julia Mayer, Angela Zedda, Axel Joachim Krafft, Katherine Lindborg, Christopher Piorkowski, Thomas Gaspar

**Affiliations:** ∗ Department of Electrophysiology, Heart Center University Dresden, Dresden, Germany; † Siemens Healthcare GmbH, Erlangen, Germany; ‡ Imricor Medical Systems, Burnsville, Minnesota

**Keywords:** Ablation, Atrial flutter, Cavotricuspid isthmus, Electrophysiology, Interventional cardiac magnetic resonance

## Abstract

**Background:**

Interventional cardiac magnetic resonance (iCMR) has been established as a radiation-free alternative compared to standard fluoroscopy-guided catheter ablation for cavotricuspid isthmus (CTI)-dependent atrial flutter to image anatomy, structural alterations, and further catheter guidance.

**Objective:**

The purpose of this study was to explore the safety, feasibility, and efficacy of CTI ablations performed completely in the iCMR suite using active catheter imaging.

**Methods:**

Consecutive patients underwent iCMR-guided catheter ablation for CTI-dependent atrial flutter. Procedures were performed in a 1.5-T magnetic resonance (MR) imaging unit with MR-conditional ablation catheters. Catheter guidance was achieved using active catheter imaging via integrated MR receive tip coils. Acute success, periprocedural complications, and short-term follow-up were collected for further analysis.

**Results:**

All patients (N = 15; 73% male; median age 70 years; interquartile range [67–82]) achieved acute procedural success without any complication. Median procedural time was 43 minutes [33–58] with median radiofrequency delivery time of 18 minutes [12–26]. Postprocedural lesion visualization scanning was completed in a median of 32 minutes [10–42]. None of the patients with 6-month follow-up had atrial flutter recurrence.

**Conclusion:**

In the iCMR suite, CTI-dependent atrial flutter ablation could be achieved safely using active catheter imaging without any complication. It further allows detailed anatomic visualization of the CTI, intraprocedural lesion visualization, and exclusion of pericardial effusion.

**Key Findings**

▪ Real-time magnetic resonance (MR)-guided ablation procedures for atrial flutter can be safely and successfully performed completely in the interventional cardiac magnetic resonance (iCMR) environment.
▪ Real-time MR-guided ablation procedures can be completed using active catheter tracking, without the need for mapping or navigation software enhancement.
▪ Real-time MR imaging provides additional information (eg, anatomic characteristics, acute lesion assessment, detection of procedure-related complications) to guide physicians in therapy delivery.
▪ This procedure represents a radiation-free ablation method.

## Introduction

Radiofrequency catheter ablation (RFCA) is the first-line therapy in patients with cavotricuspid isthmus (CTI)-dependent atrial flutter.^1^^,^^2^ In the vast majority of clinical routine procedures, RFCA for CTI-dependent atrial flutter is conducted in the electrophysiology (EP) laboratory under the guidance of fluoroscopy without a 3-dimensional (3D) mapping system. However, this standard procedure is associated with radiation exposure and suboptimal soft tissue visualization.

A decade ago, magnetic resonance (MR)–compatible ablation catheters became available, enabling the possibility of intraprocedural anatomic visualization and zero radiation exposure.

Previous studies established the efficacy and safety of procedures for CTI-dependent atrial flutter ablation in which catheter tracking was conducted using a 3D mapping system, which requires a time-consuming 3D MR prescan.^3^, ^4^, ^5^, ^6^, ^7^ The largest study (n = 30) demonstrated similar safety and success rates after 90 days compared to conventional fluoroscopy-guided procedures.^7^ However, this study used a compatible 3D mapping system, and the procedures were initiated in a standard EP laboratory and patients were transferred to the interventional cardiac magnetic resonance (iCMR) laboratory.

For the procedures performed in this analysis, we used a method to actively image and track the catheter using MR receive coils integrated into the catheter tip.^8^ This allows operators to visualize and manipulate MR-compatible catheters within the vessels and cardiac chambers in real-time MR images.

To further evaluate the safety, feasibility, and short-term outcomes, the current study was conducted in patients who underwent the entire RFCA procedure under MR guidance using active catheter imaging.

## Methods

### Study design

An investigator-initiated, prospective, nonrandomized study was conducted at Heart Center Dresden, Germany, between February 2020 and May 2021. The study was approved by the institutional ethical review board (EK 284092012), and all participants provided written informed consent. The research reported here adhered to the Helsinki Declaration as revised in 2013. Consecutive patients were enrolled for CTI-dependent atrial flutter ablation.

### Setup of iCMR suite

**Figure 1:**
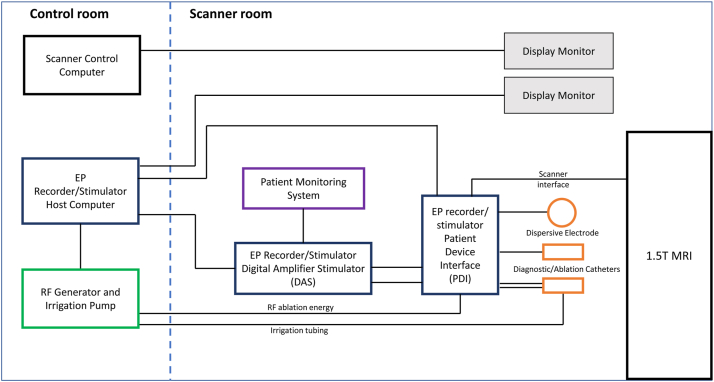
Interventional cardiac magnetic resonance suite block diagram. EP = electrophysiology; MRI = magnetic resonance imaging; RF = radiofrequency.

The setup of the iCMR suite to perform MR-guided ablation procedures without a 3D mapping system is illustrated in Figure 1. The iCMR suite is divided into 2 rooms: a control room and a scanner room (with waveguides in the radiofrequency [RF] shield to allow cabling between components in each room). MR-guided ablation procedures are performed in a conventional 1.5-T magnetic resonance imaging (MRI) unit (MAGNETOM Aera, Siemens Healthcare, Erlangen, Germany). Two MR-conditional, open-irrigated, steerable, ablation catheters (Vision-MR Catheters, Imricor Medical Systems, Burnsville, MN) with fiberoptic temperature sensing and integrated MR receive tracking coils are connected to an EP recorder and stimulator system (Advantage-MR, Imricor Medical Systems). The Advantage-MR system consists of a patient–device interface and a digital amplifier stimulator located in the scanner room and a host computer placed in the control room. It provides pacing and recording capabilities, integrates RF energy for therapy delivery, and integrates external electrocardiographic patient monitoring data for display. An RF generator and irrigation pump (IBI-T11, Abbott, Plymouth, MN) is placed in the control room and connects to the ablation catheter via the patient–device interface for RF energy and irrigation. An MR-conditional patient monitoring system (Invivo Expression Patient Monitor, Philips, Amsterdam, Netherland), located in the scanner room, provides noninvasive blood pressure, O_2_ saturation, and electrocardiographic monitoring during the procedure. The medical staff communicates via a wireless optoacoustic headphone system (IMROC, Opto-acoustics, Moshav Mazor, Israel).

A predesigned safety protocol was tested in case of emergency. This safety protocol included access to an external defibrillator (available outside of the scanner room within 30 seconds) and a standard EP laboratory blocked during procedures in case of pericardial effusion. The time for transferring a completely monitored, deep-sedated patient from the iCMR to the EP laboratory was <3 minutes in simulations.

### Patient preparation

All procedures were performed with patients in deep analgosedation, by clinical routine. In each patient, groin punctures were performed in the MR scanner. Two 9F MR-compatible catheters were introduced through 11F sheaths (Radifocus Introducer II, Terumo, Tokyo, Japan).

### Active catheter imaging and iCMR scanning during the ablation procedure

**Figure 2:**
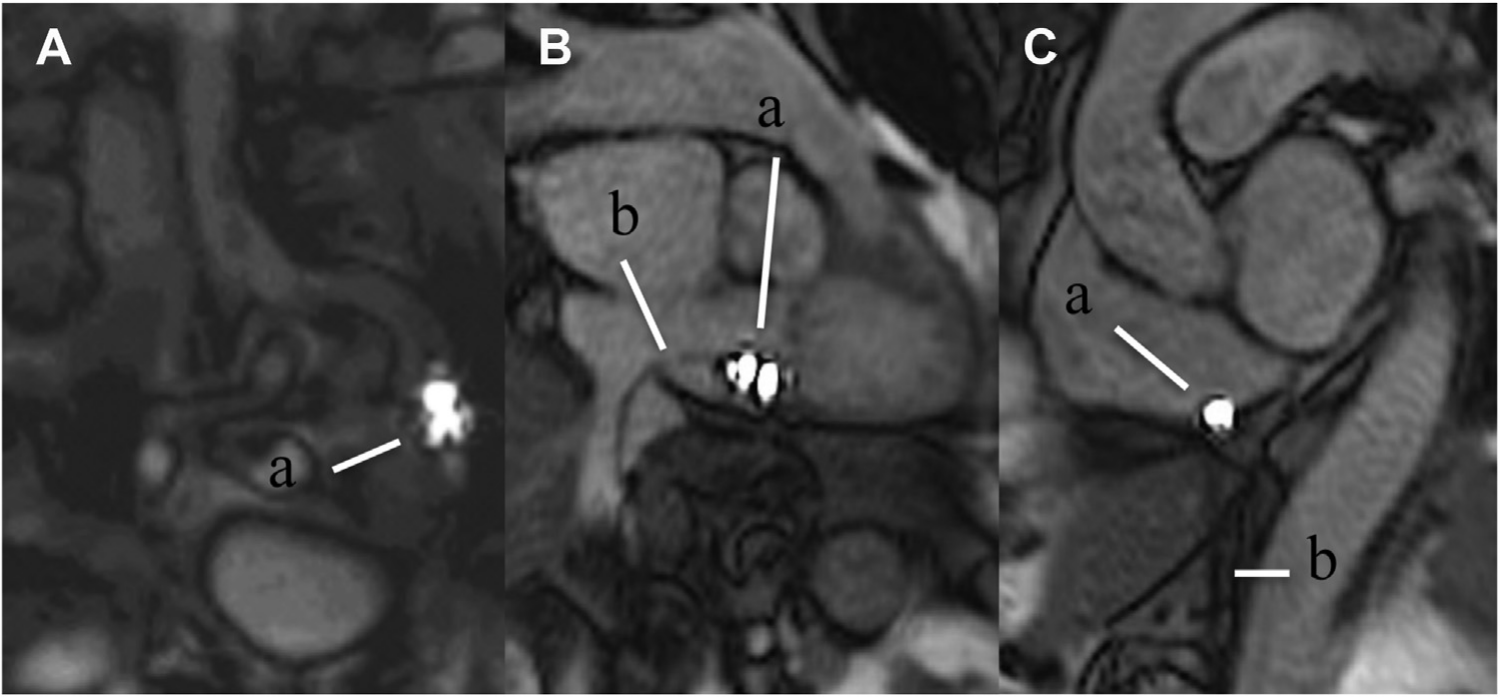
Active catheter imaging of the ablation catheter tip. Catheter imaging navigating using interactive real-time balanced steady-state free precision sequences: active imaging of the distal and proximal receive coils in the catheter tip *(a)* and passive imaging of the catheter shaft *(b)*. **A:** Ablation catheter tip in the left vena iliaca externa, guiding the catheter to the right atrium. **B:** Right anterior oblique view with the ablation catheter tip in the right atrium. **C:** Left anterior oblique view of the ablation catheter tip in the right atrium.

All CMR scans were conducted in a 1.5-T system (MAGNETOM Aera, Siemens Healthcare) with an 18-channel body array and a 32-channel spine array coil. Two of the same MR-compatible catheters were used to perform the procedures; one was connected to an RF generator and irrigation pump to serve as the ablation catheter, and the other served as a diagnostic catheter. The catheter incorporates 2 gold electrodes at the distal tip for pacing and recording electrogram signals. RF energy is delivered through the distal 3.5-mm gold-tip electrode containing 6 irrigation ports to cool the tip. The catheters are tracked and visualized via 2 MR receive coils that are integrated into the tip of the catheter and connected to the MR coil interface. These 2 miniature receive coils appear as 2 bright spots of high intensity when combined with anatomic images from the local surface imaging coil. This method of visualizing a catheter and tracking its movement throughout the anatomy is referred to as active catheter imaging (Figure 2 and Supplemental Video 1).

At the beginning of the procedure, the table position was set at the pelvis. The catheter was constantly tracked from the femoral puncture to the right atrium. When the catheter tip reached the border of the field of view, active tracking was stopped. The table was moved to the heart position. Interactive real-time balanced steady-state free precision (bSSFP) tracking sequences were used for intracardiac catheter visualization, with repetition time 4 ms; echo time 2 ms; flip angle 70°; resolution 2 × 2 × 10 mm; field of view 260 × 260 mm; matrix 95 × 128 pixels; bandwidth 1000 Hz/pixel; frame rate 6 pictures per second; tracking flip angle 7°; slice following sensitivity 1 mm; and background suppression 1 mm. For visualization of the catheters in the vena iliaca and inferior vena cava (IVC), a table position was defined on the pelvis and the catheter MR receive coils were localized by manually adjusting the sagittal image plane.

After placement of the 2 MR-compatible catheters in the IVC, a fast transversal T2-weighted bSSFP was applied. Standard projections, which typically are applied in the EP laboratory (ie, right anterior oblique [RAO] and left anterior oblique [LAO]), were reconstructed to provide views vertically and along the CTI, respectively. RAO and LAO projections were adapted in the interactive real-time bSSFP sequence, which was modified using a slice thickness of 10 mm and reduced field of view in phase encoding direction (∼80%) to achieve shorter scan times. An additional transversal view was created for visualization of the coronary sinus. During the EP procedure, image planes were chosen based on the discretion of the operator.

After placement of 1 MR-compatible catheter in the coronary sinus as a diagnostic catheter, the other MR-compatible catheter was placed at the CTI and served as an ablation catheter. The optimal ablation line was adjusted through a special process of fine visualization (fine-tuning) of the inferior tricuspid valve and eustachian valve using a reduced slice thickness (5 mm) and further confirmed under real-time tracking during ablation. By manipulating the ablation catheter in the RAO and LAO views, the best catheter starting position was found. Direct visualization of the eustachian valve and pouch was possible so that the catheter was steered to have best tissue contact. RF energy was delivered at 50 W and irrigation flow rate of 17 mL/min. Successful bidirectional isthmus block was confirmed by wide local double potentials and differential pacing at the coronary sinus ostium and the anterolateral CTI.

### Acute lesion assessment

**Figure 3:**
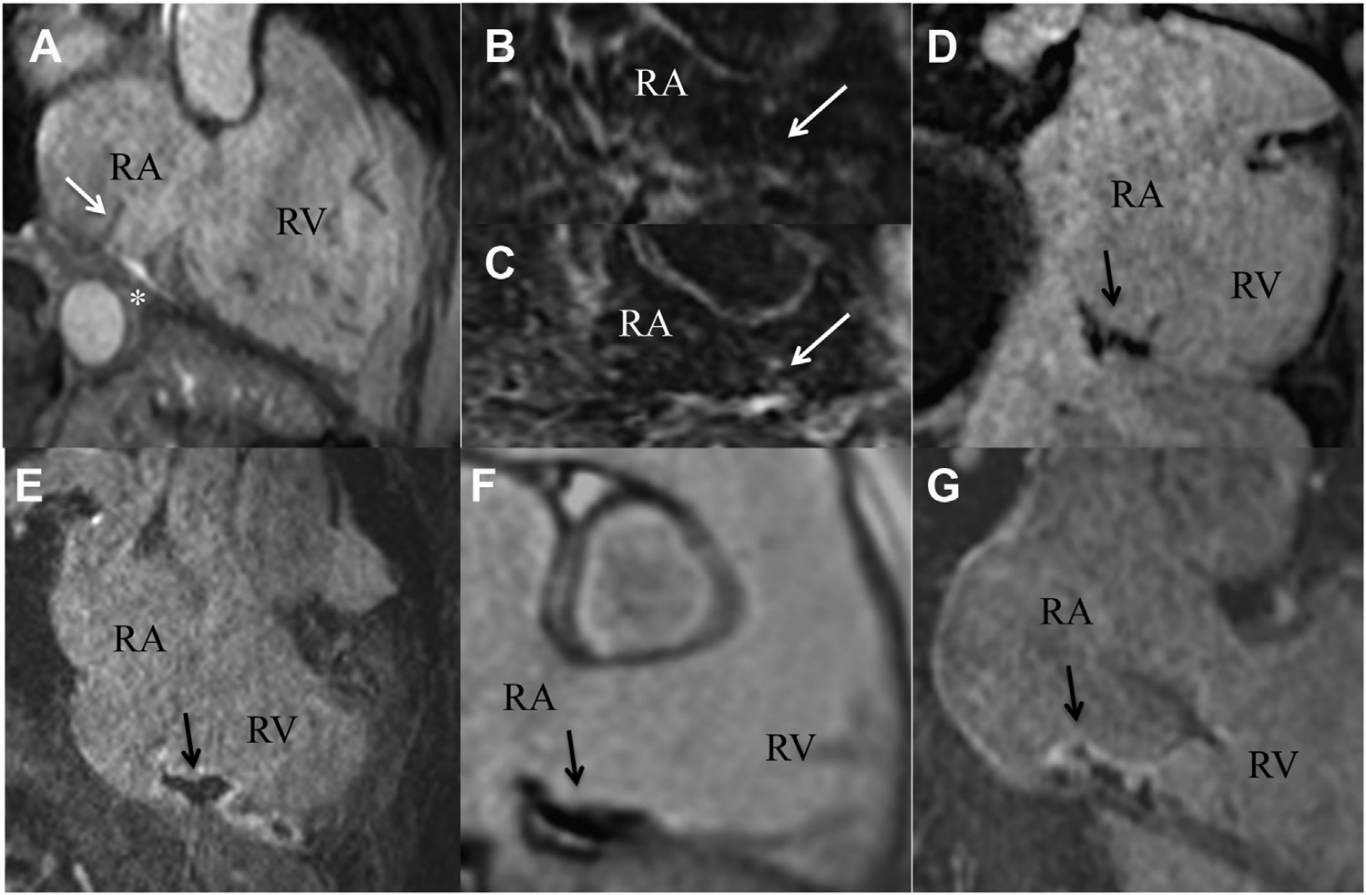
Acute lesion after radiofrequency ablation of the right cavotricuspid isthmus. **A:** Balanced steady-state free precision sequence image of the cavotricuspid isthmus (CTI) immediately after ablation. *White asterisk* indicates pericardial effusion. *White arrow* indicates a prominent eustachian valve. **B, C:** T2-weighted images preablation **(B)** and postablation **(C)** showing signal intensity enhancement of the isthmus line (*white arrows*). **D:** Noncontrast enhanced T1-weighted image of the CTI depicts acute necrotic lesions as signal intensity loss (*black arrow*). Inversion time was adjusted to suppress the acute lesion. **E:** Postcontrast early enhancement image shows hypoenhanced myocardium localized at the CTI, known as a microvascular obstruction as an acute ablation lesion sign. **F:** Phase-sensitive inversion recovery image depicts acute ablation lesion in terms of black, hypoenhanced myocardium. **G:** Postcontrast late gadolinium enhancement images led to partially enhanced radiofrequency ablation lesions (*white edge*) with black necrotic core (*black arrow*).

After the confirmation of bidirectional block, in a transversal pseudo-4CH view a triggered bSSFP cine image (resolution 1.8 mm × 1.8 mm × 6 mm; matrix 156 × 192 mm) was used for exclusion of pericardial effusion. Furthermore, in the previously adjusted RAO view for optimal visualization of the CTI ablation line, T2-weighted images (navigator-gated, resolution 1.3 mm × 1.3 mm × 6 mm; repetition time 1500 ms; echo time 70 ms) and a noncontrast 3D T1-weighted pulse sequence (TWILITE: 3D-navigator gated, resolution 1.1 mm × 1.1 mm × 1.3 mm; matrix 208 × 256 pixels) were conducted aiming to establish acute lesions,^9^ using a modified inversion time. As an example, in Figure 3, acute lesions were suppressed, and blood signals were enhanced.

After administration of 0.2 mmol/kg of intravenous gadolinium chelate contrast agent (Dotarem, Guerbet, Sulzbach, Germany), early and late enhancement images of 2D/3D segmented inversion-recovery spoiled echo gradient sequences (3D-navigator-gated, resolution 1.3 mm × 1.3 mm × 2.5 mm; matrix 256 × 256 pixels) were confined in the vicinity of the CTI ablation line, aiming to reduce the time of postprocedural scanning. Catheters were placed in the IVC during imaging to avoid artifact. Microvascular obstruction (MVO) was defined as a central hypoenhanced region within hyperenhanced tissue on late enhancement 6 minutes after contrast administration.

### Statistical analysis

Due to limited sample size, all continuous data are presented as median [interquartile range]. Categorial variables are given as number (percentage). Statistical analysis was performed using SPSS 19 (IBM, New York, NY).

## Results

### Patient characteristics

Fifteen consecutive patients (median age 70 years [67–82]; 11 male [73%]) were enrolled in the study between February 2020 and April 2020. Follow-up visits were conducted for 6 months postprocedure. Detailed baseline characteristics are given in Table 1.

**Table 1:** Patient characteristics (N = 15)

Age (y)	70 [67–82]
Male	11 (73)
Body mass index (kg/m^2^)	27 [25–32]
Hypertension	13 (87)
Diabetes mellitus	4 (27)
Coronary artery disease	6 (40)
Tachycardia-induced cardiomyopathy	5 (33)
LVEF at baseline (%)	47 [29–59]
LVEF at 6-month follow-up (%)	52 [45–59]
CHA_2_DS_2_-VASc score	4 [3–5]
Antiarrhythmic drugs Beta-blocker Verapamil Flecainide Amiodarone	11 (73) 6 (40) 0 (0) 4 (27)

Values are given as median [interquartile range] or n (%).

LVEF = left ventricular ejection fraction.

### Procedural characteristics, periprocedural complication, and follow-up

Bidirectional block of CTI ablation lines was achieved in all 15 patients. No periprocedural complications occurred. Median time from femoral vein puncture to achievement of bidirectional CTI block was 43 minutes. Median time from femoral vein puncture to access of the right atrium was 4 minutes, and catheter placement in the coronary sinus was 7 minutes. Median RF ablation time was 18 minutes. Assessment of lesion qualities was achieved by visualizing the continuity of lesions using late gadolinium enhancement. In 8 of 15 cases, MVO was evident within the ablation line. Median postprocedural scan time was 32 minutes (Table 2). Short-term follow-up of 6 months was achieved in 13 patients (1 lost to follow-up and 1 death). All were free from recurrence of atrial flutter at 6-month follow-up.

**Table 2:** Interventional CMR-guided ablation time (N = 15)

Total interventional procedure	43 [33–58]
Time to the right atrium	4 [3–5]
Time to the coronary sinus	7 [6–10]
Ablation duration	18 [12–26]
Postprocedural scan	32 [10–42]

Values are given as median [interquartile range] in minutes.

CMR = cardiac magnetic resonance.

### Anatomic characteristics

**Figure 4:**
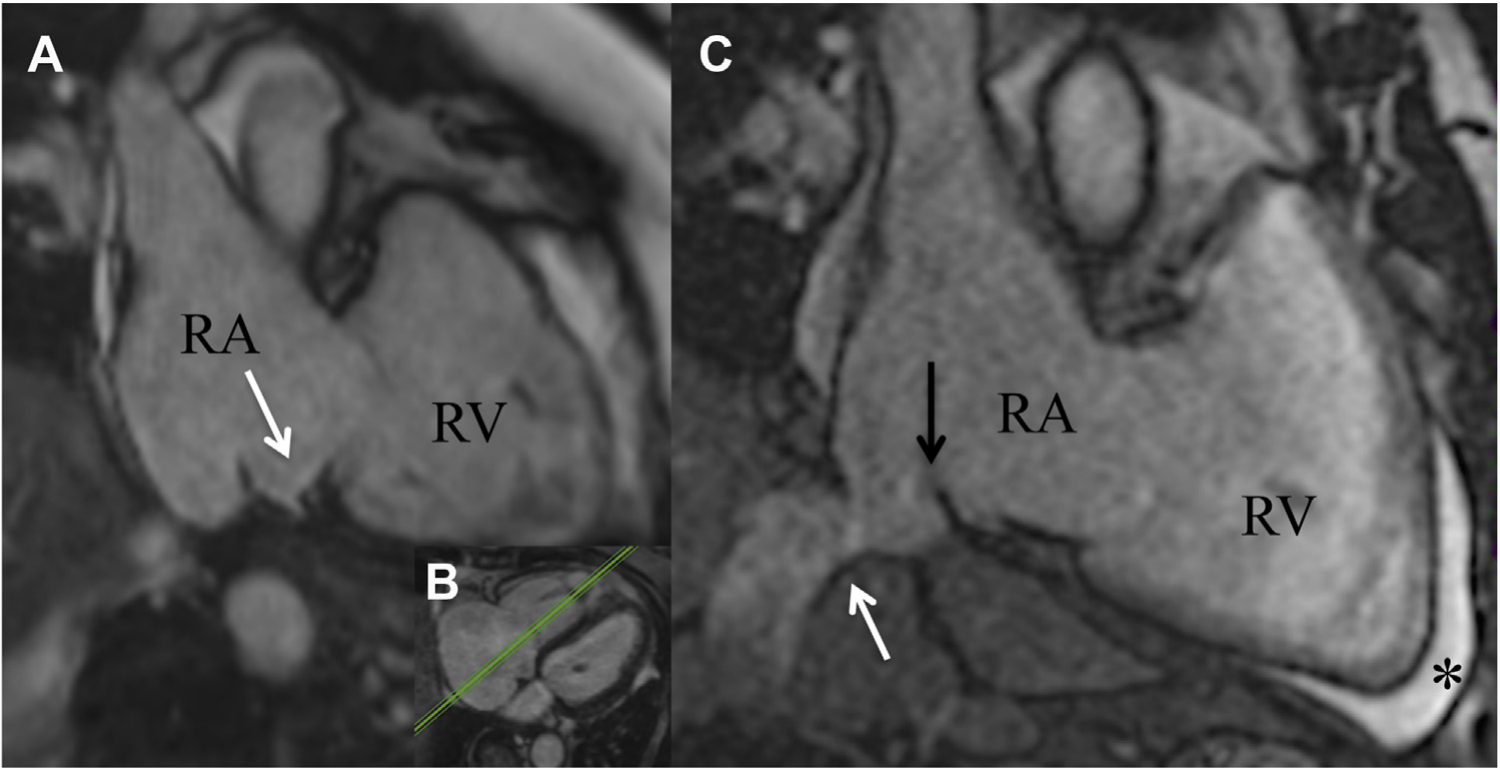
Anatomic characteristics. **A:** Septal pouch (*white arrow*) of the cavotricuspid isthmus in a balanced steady-state free precision sequence image performed during ablation remote from the septal localized coronary sinus ostium (as visualized in the transversal plane **[B]**). **C:** Kinking of the vena cava inferior junction in the right atrium (*white arrow*) and a eustachian valve (*black arrow*) was found. Anterior to the right ventricular apex, a pre-existing pericardial effusion was located (*asterisk*).

Detailed ablation-related anatomic characteristics are listed in Table 3. Unusual anatomic features were detected in 60% of patients, such as pouches remote at the CTI in 3, high eustachian valve >15 mm in 3, and kinked IVC in 3 (Figure 4). None of these findings was associated with an increase in procedural time.

**Table 3:** Anatomic characteristics (N = 15)

CTI and atrial anatomy	
Presence of septal pouch	3 (20)
Presence of eustachian valve	12 (80)
Maximal eustachian valve length (mm)	12 [10–16]
Kinking of IVC	3 (20)
Maximal systolic CTI length (mm)	22 [16–26]
Minimal diastolic CTI length (mm)	14 [9–20]
Right atrial area (mm^2^)	25 [23–28]
Left atrial area (mm^2^)	28 [24–30]

Values are given as n (%) or median [interquartile range].

CTI = cavotricuspid isthmus; IVC = inferior vena cava.

## Discussion

This is the first real-world, postmarket, prospective study completing a full procedure of zero-fluoroscopic, active catheter imaging–guided catheter ablation in the iCMR suite without a 3D mapping system. The workflow of MRI-guided conventional catheter ablation, which was developed and tested over the course of the study, has been proven to be able to reach the study endpoints with regard to safety and feasibility.

### Previous studies of CTI-dependent atrial flutter ablation

CTI-dependent atrial flutter ablation is a relatively simple procedure with the lowest intervention-related complication rate, which allows it to serve as a proper candidate for ablation using novel techniques. As the first step in moving ablation procedures into the MR environment, it allows physicians to conduct procedures comfortably and to gain confidence constantly in a novel environment.

Previous premarket studies of MRI-guided ablation were conducted with a combination of preparation in the standard EP laboratory and ablation in an iCMR laboratory.^3^, ^4^, ^5^, ^6^, ^7^ In the current setting, femoral vein access, catheter placement, and further ablation were performed entirely in the iCMR laboratory. It brings several benefits to the field, including a zero radiation exposure procedure and detailed real-time imaging on targeted anatomies. In addition, the ability to perform all steps from femoral puncture up to ablation in a single room may help to reduce the risk of infection for patients.

To date, as a standard procedure, CTI-dependent atrial flutter ablation in clinical routine has been conducted in a standard EP laboratory using conventional ablation catheters without 3D mapping systems. Large studies of catheter ablation of CTI-dependent atrial flutter reported that total procedural time, X-ray exposure time, and time of ablation were 75 [55–103] minutes, 13 [7–28] minutes, and 19 [7–28] minutes, respectively.^10^ In the current study, the durations of each step were comparable to those of conventional ablation procedures in a standard EP laboratory. There is no evidence that the novel real-time active catheter imaging technique negatively affected catheter placement, manipulation during ablation, and further arrhythmic outcomes.

### Individualized CTI lines and related anatomic structures in iCMR

**Figure 5:**
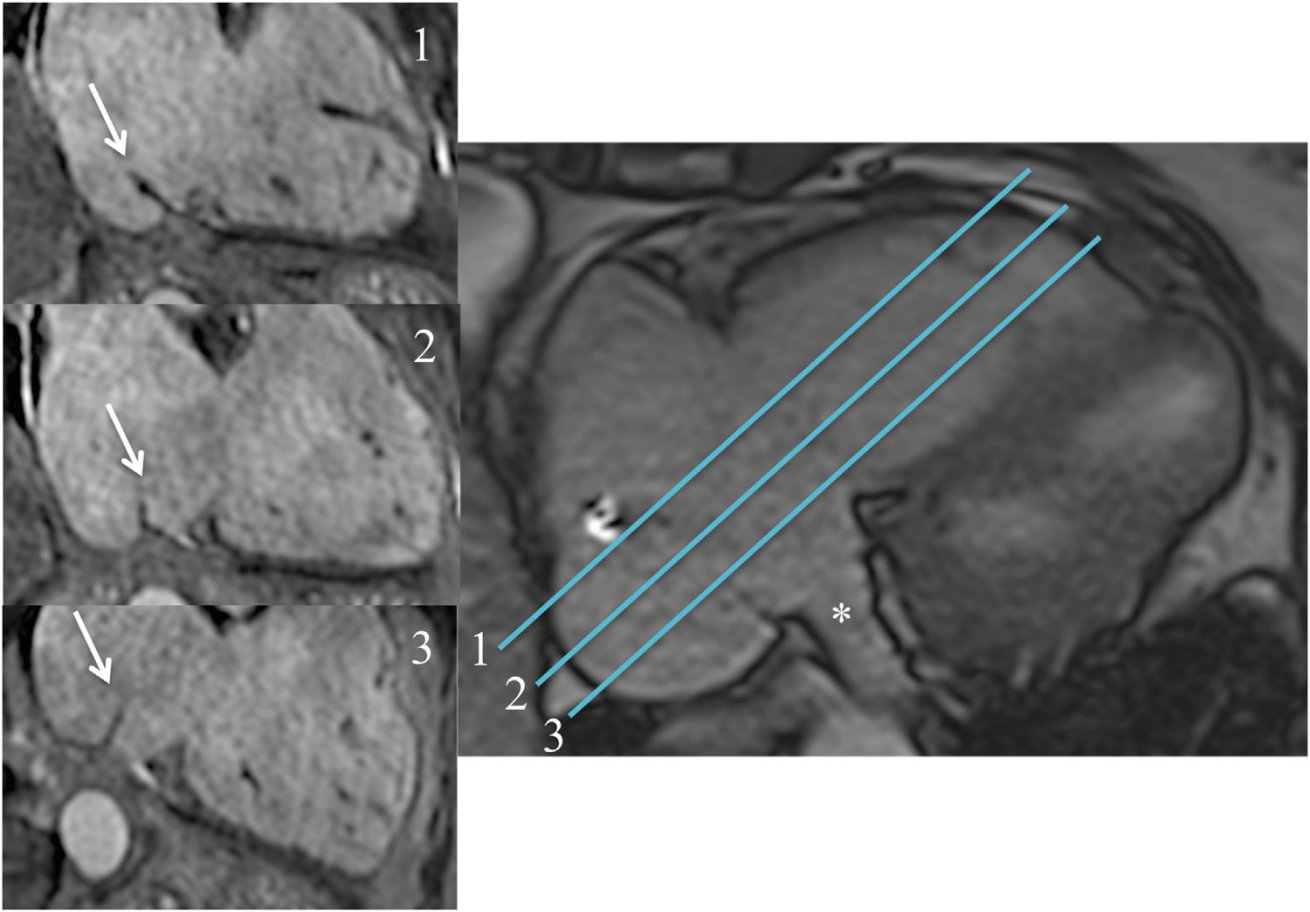
Eustachian valve imaging. The length and angle of the eustachian valve (*white arrow*) and consecutively the accessibility of the cavotricuspid isthmus (CTI) depends on a lateral (*1*), mid (*2*), or septal (*3*) slice orientation in the transversal plane. The CTI line in slice 2 is deemed optimal for ablation because of a shorter length of eustachian valve and a sufficient distance from the coronary sinus ostium (*white asterisk*).

Previous studies reported that presenting anatomic abnormalities (eg deep pouches, higher eustachian valve, longer CTI) extend procedural time and increase complications in nearly 11% of patients undergoing a CTI-dependent atrial flutter ablation procedure.^11^, ^12^, ^13^, ^14^ However, in the current study, none of the anatomic abnormalities was associated with an increase in procedural time. We speculate that the nonincreased procedural time in the MRI-guided ablation procedure could be explained by the ability to predefine the ablation lines according to the patient-specific anatomic information available from iCMR imaging. In this cohort, we identified anatomic structures such as pouches, eustachian valves, and kinked IVC in a significant number of patients, structures that may decrease the accessibility of CTI with the ablation catheter without knowledge of the detailed anatomy (Table 3 and Figure 4). Furthermore, CTI length can be quantified to optimize the ablation line and further assist the detection of conduction gaps. The anatomic features identified in real time during the procedure assist physician operators in planning and directing therapy delivery to create a complete CTI ablation line. Additionally, transversal planes of the right atrial isthmus are obtained and sliced orthogonally so that an optimal isthmus line can be identified (Figure 5).

MRI delivers excellent soft tissue contrast, so it is the method of choice for acute lesion assessment. We believe that the long-term durability of ablation lines depends on accurate transmural lesion formation. However, MR left ventricular infarct studies have shown that acute edema can resolve, and transmurality of infarct lesions cannot be assessed not until several weeks later. There is evidence that MVO is a parameter that represents more severe damage to the myocardium. In our study, MVO was present in 8 of 15 cases. Whether MVO can be a marker of long-term durability needs to be addressed in studies with longer follow-up.

Fluoroscopy-free electrophysiological procedures have evolved.^15^ In our opinion, iCMR provides additional information for the operator because of its superior anatomic visualization, substrate imaging, lesion assessment, and immediate imaging of complications. The current MR-compatible equipment for ablation procedures is limited but sufficient for CTI ablations.

Once devices to access and support left-sided procedures, including transseptal access devices, have been developed, the benefits of iCMR can be demonstrated more significantly in complex procedures including substrate-related rhythm disturbances. In our view, direct visualization of the ventricular substrate and immediate proof of adequate lesion formation would be beneficial in such procedures.

### Study limitations

This was a prospective, nonrandomized study with a relatively small sample size for optimizing the workflow of CTI ablation in iCMR and without a control group. Because of the lack of treatment consensus in iCMR, we decided to use a nonrandomized study design to describe the safety, feasibility, and clinical outcomes using this novel workflow. Further evidence-based validation using a control group, large sample size, and randomized design is necessary.

## Conclusion

This study presents initial, real-world results achieved with a clinically acceptable workflow for CTI-dependent atrial flutter ablations performed entirely in the iCMR laboratory using active catheter imaging without the need for a 3D mapping system. This is the first step in moving entire ablation procedures from the standard EP laboratory to the iCMR laboratory as part of clinical routine. It provides a zero radiation exposure procedure environment and further facilitates ablation procedures through real-time imaging of related cardiac anatomy, the ability to evaluate the quality of ablation lesions, and the screening of periprocedural complications.

## Appendix Supplementary data

Supplemental Video 1 The supplemental video shows an example of the MRI guided catheter imaging during advancement to the heart, coronary sinus cannulation and CTI ablation.
